# Bacterial colonization, species diversity and antimicrobial susceptibility patterns of indwelling urinary catheters from postpartum mothers attending a Tertiary Hospital in Eastern Uganda

**DOI:** 10.1371/journal.pone.0262414

**Published:** 2022-01-10

**Authors:** Ashley Winfred Nakawuki, Rebecca Nekaka, Lydia V. N. Ssenyonga, George Masifa, Dorreck Nuwasiima, Julius Nteziyaremye, Jacob Stanley Iramiot

**Affiliations:** 1 Faculty of Health Sciences, Department of Nursing, Busitema University, Mbale, Uganda; 2 Faculty of Health, Department of Community and Public Health, Busitema University Sciences, Mbale, Uganda; 3 Mbale Clinical Research Institute, Mbale, Uganda; 4 Faculty of Health Sciences, Department of Obstetrics and Gynaecology, Busitema University, Mbale, Uganda; 5 Faculty of Health Sciences, Department of Microbiology and Immunology, Busitema University, Mbale, Uganda; Nitte University, INDIA

## Abstract

**Background:**

Postpartum urinary Catheter-Related Infections (CRIs) are a significant cause of maternal sepsis. Several studies done have reported the presence of mixed populations of bacteria with a significant increase in Extended-Spectrum Beta-Lactamase (ESBL) Enterobacteriaceae spps, Methicillin-Resistant Staphylococcus aureus (MRSA), Multi-Drug Resistant (MDR) bacteria in urine and blood cultures of catheterized patients despite the use of prophylactic antibiotics. This study aimed at determining the bacterial species diversity and susceptibility patterns of indwelling urinary catheters from postpartum mothers attending Mbale Regional Referral Hospital (MRRH).

**Methods:**

A cross-sectional study employing quantitative and qualitative was carried out in MRRH among postpartum mothers with urinary catheters and their care-takers. The purposive non-random sampling strategy was used to collect data using an interviewer-administered questionnaire for the quantitative data collection and in-depth interviews for qualitative data collection. All the data collection tools used were developed, pretested and validated. At the point of de-catheterization, Catheter tips from enrolled participants were cut about 2-3cm below the balloon aseptically into test-tube containing peptone water, sonication technique employed, and incubation done 24hours then cultured to ensure phenotypic identification. An antibiotic sensitivity test was performed using the disc diffusion method following Clinical and Laboratory Standards Institute (CLSI) guidelines. Quantitative data collected was entered in Microsoft Excel and then exported to STATA14 for statistical analysis. Thematic analysis was used to analyse and organise qualitative data by an inductive coding method using Nvivo 12 software.

**Results:**

In this study, 208 postpartum mothers participated, the majority of whom were caesarean section mothers of age range 20–24 years and 17 care-takers with a median age of 32 years. The prevalence of catheter tips bacterial colonisation was 98% despite 88.5% of the participants being on broad-spectrum antibiotics. The average duration of catheterisation was 2 days. All bacteria isolates were potential uro-pathogens with a mean occurrence of 2 bacteria species in each urinary catheter tip. The rates of MDR to commonly used antibiotics were high. The urinary catheter size of greater than F14 and duration of catheterization greater than 2 days were significantly associated with the number of bacterial species isolated from each sample. The maintenance care and knowledge of care-urinary catheter care among the care-takers was found sub-optimal.

**Conclusion:**

There was a high prevalence of catheter colonisation with bacterial spps diversity averaging 2 spps per sample despite use of broad spectrum antibiotics. The MDR rates were high, which calls for routine culture and sensitivity. Health workers practicing obstetric medicine need to pay attention to catheter sizes during catheterisation and its duration. Health education should be part of antenatal and postnatal care education.

## Background

Postpartum urinary Catheter-Related Infections (CRIs) are a significant cause of maternal sepsis [1]. Sepsis alongside haemorrhage and hypertensive disorders constitute an infamous triad that causes significant maternal and perinatal morbidity, with >90% of these occurring in low-income countries. Globally, Maternal mortality ratio attributed to sepsis is 10.7% [2]. Uganda has one of the highest maternal mortality rate (MMR) at 336 per 100.000 live births [3]. Sepsis in Uganda has emerged as a leading cause of mortality [4]. This is a direct threat to the achievement of sustainable development goal (SDG 3) [5].

Pregnant women are more predisposed to UTI due to physiological and anatomical changes [6]. Studies done in eastern Uganda have documented significant bacteriuria among this population [7,8]. Moreover, whereas urinary catheterisation is an added risk to sepsis [9,10], intrapatum catheterisation is at times inevitable as it serves as prophylaxis against vesicle damage and is as well significant during investigations and therapeutic purpose. Furthermore, intrapartum complications such as caesarean section, obstructed labour, extensive perineal tears that warrant long periods of catheterisation pose an additional risk to urinary CRIs [13]. A study carried out amongst postpartum mothers in a tertiary centre in Western Uganda reported 14% overall prevalence of postpartum UTIs, with 76% CAUTI and 90% occurring in caesarean section mothers [11]. Although CAUTI is the most common adverse event associated with indwelling urinary catheter use [12,13]: Catheter-associated urinary tract infection is rarely symptomatic, only 3% will develop bacteraemia with a urinary isolate [14]. However, given the high frequency of catheterization in Obstetrics practice, this presents a significant challenge. Furthermore, research has reported that despite these mothers being on prophylactic antibiotics, resistance is common [11]. Several studies done have reported the presence of mixed populations of bacteria in urine and blood cultures of catheterized patients, with a significant increase in Extended-Spectrum Beta-Lactamase (ESBL) Enterobacteriaceae spps, Methicillin-Resistant Staphylococcus aureus (MRSA), and Multi-Drug Resistant (MDR) bacteria [11,15,16].

Whereas Catheter-related infections are preventable by measures put in place to prevent device-related HCAI, low-income countries have challenges implementing them. These countries such as Uganda, face challenges that include inadequate environmental hygiene, poor infrastructure, insufficient equipment, understaffing, and overcrowding [9,17].

Several other factors such as duration of catheterization, meatal colonisation with uropathogens, microbial colonisation of drainage bag, catheter insertion outside operation room, catheter care violations, absence of the use of a drip chamber, diabetes mellitus(DM), and rapidly fatal underlying illness such as Human Immune deficiency Virus/Acquired Immune Deficiency Syndrome(HIV/AIDS) have also been reported to increase the risks of catheter-associated bacteriuria among postpartum mothers [12,18–20].

Among postpartum mothers, CRIs have been associated with consequences such as slowing the mother’s recovery resulting in prolonged hospital stay, early-onset neonatal sepsis and also indirectly affect new-born wellbeing, causing difficulties such as in breastfeeding and by interfering with mother and child bonding [21]. Other consequences include increased antimicrobial resistance, high economic burden, more severe complications being bacteremia, upper urinary tract infection, endometritis, and excess mortality [22]. This study aimed to determine colonisation, the bacterial species diversity and susceptibility patterns in indwelling urinary catheters of postpartum mothers attending Mbale Regional Referral Hospital.

### Justification

Catheter-associated infections contribute to maternal and perinatal morbidity and mortality especially in low-income countries like Uganda. However, CRIs are preventable probably by adjusting the days of catheterization, catheter, and drainage care practices, knowing which organisms and what they are sensitive to, and thus giving appropriate antibiotics. The study will contribute to this body of knowledge and influence practice.

## Materials and methods

### Study design and population

A cross-sectional study design using both quantitative and qualitative data collection methods was carried out. The study population was postpartum mothers with indwelling urinary catheters and admitted to Mbale Regional Referral Hospital.

### Study area

The study was carried out in the Department of Obstetrics and Gynaecology, Mbale Regional Referral Hospital. It serves as a teaching hospital for Busitema University, Mbale School of clinical officers, and other nursing schools as well as an internship centre for medical interns. The Obstetrics and Gynaecology department has three major wards High Dependency Unit, Postnatal, and gynaecology ward. The wards admit gynaecology patients, postpartum mothers, and mothers who have lost their babies. On average, 30–35 mothers deliver per day, of whom 5–6 mothers deliver by emergency caesarean section. Every Tuesday, about 3 elective caesarean section cases are done. This brings the average number of postpartum mothers to 210–245 altogether per week out of which 40 deliver by caesarean section.

#### Sampling technique

A purposive non-random sampling strategy was used to select the participants for this study.

#### Inclusion criteria and exclusion criteria

Postpartum mothers with indwelling urinary catheters and persons in charge of urinary catheter care (Care-takers/mothers) of catheterized postpartum mothers were included in the study. Postpartum mothers with indwelling urinary catheters who were de-catheterized in absence of the researcher or prior to consenting for the study were excluded.

### Data management and analysis

Data was collected using pretested questionnaires, document review, in-depth interviews and bacterial culture and susceptibility testing. An interviewer-administered questionnaire was used to capture socio-demographic, obstetric/gynaecological patient details, duration of stay of the catheter, and indication for placement. In the in-depth interviews, persons (care-taker /mother) who took charge of the care of the urinary catheters and drainage bag participated in a discussion where open-ended questions were asked and responses were required from them. A developed interviewer guide which had open-ended questions and probes were used to assess the knowledge and behaviour practice during catheter care. Each participant took 20–30 minutes. Care-takers were interviewed in a language they best understood, and then the information was transcribed to generate meaning-full themes. Patient charts were reviewed to identify vital information like diagnosis made, current treatment, and indication for catheterization to help enrich associated factors. All data collection tools were specifically developed for this study.

Data from the questionnaires and patient records were analysed by descriptive statistics using STATA version 14. The data was presented in frequencies, percentages, and measures of central tendencies obtained were displayed mainly on the tables, graphs, percentages, and pie charts.

For the in-depth interviews, an inductive analysis approach that was entirely based on an iterative reading of data and preliminary analysis was used [23,24]. New themes emerged from the data and the data was analysed using a framework analytical approach. The steps involved in data analysis included; data familiarization, coding and identification of a thematic framework, quote sorting, thematic categorization of quotes and interpretation of outcomes.

The principle investigator initially familiarized with the data by reading and rereading the transcripts which ensured easy accessibility to the transcripts at a later stage. The in-depth interviews were coded independently to ensure credibility and trustworthiness, during familiarization and coding new codes emerged and categories that led to an iterative process. In the transcripts several quotes were identified with several themes and the process was reiterated to avoid overlooking any emerging themes.

NVIVO software version 12 (QSR International, Burlington, Massachusetts) was used to classify the nodes as free and tree nodes. Tree nodes refer to codes that are organized hierarchically into categories and subcategories, while free nodes are emergent themes that are not attached to a pre-existing tree node. The nodes developed were used to code the transcripts inductively following a process of constant comparison between the emerging themes/codes and pre-existing codes. Any emergent code was added as a free node or attached to a tree node according to its place in the initial thematic framework. Salient quotes were noted. Coding density, which refers to the strength of association between themes, was used to identify recurrent themes. The themes with the highest coding density were categorized as major themes and others as minor themes. Based on this, a final thematic model was developed. Qualitative data was presented in phrases under generated themes and sub-themes.

### Sample collection

The postpartum mothers with indwelling catheters who gave consent were included in the study. The urinary catheters were removed as directed by the reviewing medical personnel during the ward-rounds, and its tip was cut aseptically about 2-3cm below the balloon point with a disinfected pair of scissors into peptone water. This was then transported to Busitema University Faculty of Health Sciences Microbiology laboratory where cultures were done to identify the bacterial species and antimicrobial susceptibility patterns to the commonly used antibiotics. Efforts were made to ensure that the sample reaches the microbiology laboratory in less than 1hour. Broth with catheter tip was transported within a cooler box.

### Culture of catheter tips and purifying of isolates

Catheter tips were incubated in peptone water for 24hours at a temperature of 37°C. A micro-pipette was used to draw 25ml from each of the samples then inoculated on to MacConkey agar and chocolate agar. The inoculum was spread evenly using a wire loop on the plate with culture media. Cultures were then incubated at 37°C for 24 hours after which plate reading was done to identify the bacteria growth characteristics on each plate. Purity was done on MacConkey agar to isolate organisms with different growth characteristics and incubated for 24 hours. Plates with pure growth were gram stained, microscopy, and different biochemical tests such as oxidase test, Simon’s citrate, indole, Sim’s motility test, catalase, and coagulase test were done to identify bacterial organisms. Also, Brilliance Chromogenic agar and analytical profile index (API) were used as confirmatory tests.

### Antibiotic susceptibility testing

Antimicrobial sensitivity tests were done on pure colonies of each species to commonly used antibiotics using a Kirby-Bauer disk diffusion method on Mueller Hinton agar plates that passed a 24-hour sterility test. The antibiotic discs used were ciprofloxacin (5μg), meropenem (10μg), cefotaxime (30μg), cefoxitin (30μg), nitrofurantoin (300μg), Trimethoprim-sulfamethhoxazole (25μg) and amoxicillin-clavulanate (30μg). Antibiotics were placed at a distance of 30mm from each other and the plates were incubated overnight at 37°C. Diameters of zone inhibition were measured using a zone inhibition ruler and recorded. The diameters were then compared with standards of inhibition according to the Clinical and Laboratory Standard Institute guidelines (CSLI, 2019) and found to be sensitive, intermediate, or resistant. Multidrug resistance was defined as one isolate being resistant to three or more classes of antibiotics tested [23,25,26].

The duration of stay of the catheter was calculated depending on the time the catheter was placed morning, evening, afternoon.

#### Quality control validity and reliability

A standardized questionnaire was used to avoid instrument bias. The questionnaire was pretested among 10 postpartum mothers that met the inclusion criteria, data collected was then reviewed for consistency and validity before the questionnaire was used for the study. The research assistants who took part in the research were trained before administering the questionnaires.

Laboratory procedures were performed by laboratory scientists under the close supervision of a clinical microbiologist to ensure quality results are obtained. Data was double entered into Microsoft excel for accuracy and reliability. ATCC 25922 E.coli was used as a control strain for quality assurance during isolation and drug susceptibility testing.

### Ethical consideration

Ethical clearance was obtained from the Mbale Regional Referral Hospital Research and Ethics Committee, the REC application number **MRRH-REC OUT 013/20**. Written informed consent was obtained from all participants in the study before data collection in line with guidelines set by good clinical practice and Uganda National Council for Science and Technology (UNCST). The participants had complete right to withdraw from the study at any time without any explanation, and without affecting their right to standard care offered in the department of obstetrics and gynaecology. Non-person identifiers were used during analysis to ensure confidentiality. The study had minimal potential risks to participants and all possible measures were taken to prevent any harm to participants during this investigation. The study had no direct benefits to participants but data collected will be used to better management of postpartum mothers with indwelling catheters. All the study’s potential benefits and risks were shared with the participants when they were requested to participate in the study.

## Results

### Demographics characteristics of postpartum women enrolled in the study

A total of 208 participants were included in the qualitative study. The mean age of study participants was 25.32(SD 5.722) years, median 25 years with a range of 17–40 years. The majority of the participants were caesarean mothers most of whom were young mothers 20–24 years. The participants that were referred from other health facilities were 79(38.9%), 164(78.9%) stay in rural areas whereas 44(21.1%) stay in urban areas, 44.7% mothers had a family income of < 200000Ugshs, 8(3.8%) participants were HIV positive, and 70(33.7%) were primiparous (Table 1).

**Table 1:** Demographic characteristic of postpartum mothers with urinary catheters that were enrolled in the study at MRRH.

Characteristic	Frequency (%)	Characteristic	Frequency (%)	Characteristic	Frequency (%)
**Age ranges**		**Education**		**Gestation age**	
15–19	40(19.2)	None	4(1.9)	preterm	22(10.6)
20–24	62(29.8)	Primary	103(49.5)	term	183(88)
25–29	58(27.9)	Secondary	79(38)	post-term	3(1.4)
30–35	36(17.3)	Tertiary	22(10.6)	**Antenatal attendance**	
>35	12(5.8)	**HIV status**		yes	205(98.6)
**Residence**		Negative	200(96.2)	no	3(1.4)
Rural	164(78.9)	Positive	8(3.8)	**Antenatal contacts**	
Urban	44(21.1)	**Referral**		<4	57(27.4)
**Marital status**		yes	79(38)	4–8.	146(70.2)
Not married	29(13.9)	no	129(62)	>8	2(1)
Married	179(86.1)	**Parity**		**Duration of catheterization**	
**Family income**		1	70(1.9)	<2 days	51(24.5)
<290,000	93(44.7)	2–4	104(50)	2-3days	135(64.9)
290,000–599,000	68(32.7)	>4	34(16.3)	≥4 days	22(10.6)
>600,000	47(22.6)	**Mode of delivery**		**History of Treatment of a UTI during pregnancy**	
**Current antibiotic use**		vaginal	36(17.3)	Yes	102(49)
yes	184(88.5)	c/s	172(81.7)	No	106(51)
no	24(11.5)				

Age in years, family income in Ugandan shillings.

### Bacterial species diversity from urinary catheters of post-partum mothers

**Fig 1:**
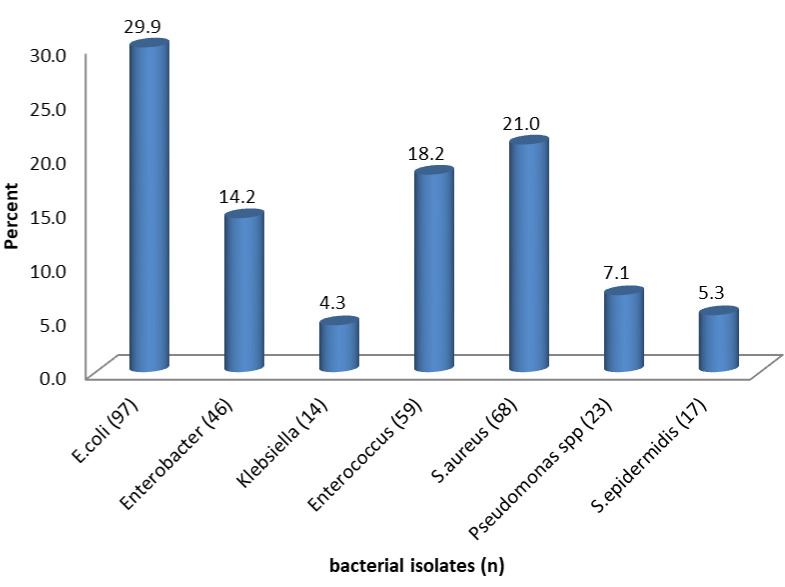
Bacterial species isolated from cultures of urinary catheter tips from postpartum mothers in MRRH.

Of the 208 samples collected, 204(98%) samples had growth and 4(2%) had no growth after 24-48hours of culture and incubation, showing catheter colonization of 98%. From 204 samples that had growth, 100(49.0%) samples yielded one isolate, 90(44.1%) had 2 isolates, and 14(6.9%) had 3 isolates. Therefore, a total of 324 isolates were obtained and identified. The number of microorganisms identified ranged from 1–3 bacteria, with an average of 2 bacterial species that were isolated from each sample. Both gram-negative and gram-positive bacterial species were isolated (Fig 1).

### Anti-microbial susceptibility of isolated bacterial species from indwelling urinary catheters of postpartum mothers

All pure bacterial isolates were tested on the commonly used antibiotics namely Amoxicillin-clavulanate 30μg, ciprofloxacin 5mcg, cefotaxime30μg, Nitrofurantoin 200μg, cefoxitin30μg, trimethoprim/sulfamethoxazole 25μg, and meropenem 10μg and in addition, Pseudomonas spps were tested on piperacillin/tazobactum 110μg. All bacteria isolated had high resistance to Sulfamethoxazole/trimethoprim, ceftriaxone, amoxicillin-clavulanate, and ciprofloxacin. The gram-negative isolates had a high sensitivity to meropenem as opposed to gram positives (Table 2).

**Table 2:** Antimicrobial susceptibility patterns of bacterial isolate of urinary catheters from post-partum women.

ISOLATE	PATTERN	AMC 30μg n (%)	CIP 5mcg n (%)	CTX 30μg n (%)	MEM 10μg n (%)	SXT 25μg n (%)	FOX 30μg n (%)	F 200 mcg n (%)	PTZ 110mcg n (%)
**GRAM NEGATIVE BACTERIA**									
*E*. *coli*	S	16(22.9)	23(31.5)	8(11.6)	54(75.0)	5(7.4)	37(88.1)	41(63.1)	
	I	22(31.4)	13(17.8)	2(2.9)	7(9.7)	0(0)	0(0)	14(21.5)	
	R	32(45.7)	37(50.7)	59(85.5)	11(15.3)	63(92.7)	5(11.9)	10(15.8)	
*E*.*aerogenes*	S	6(16.7)	9(23.1)	2(5.1)	22(59.5)	7(22.6)	20(80.0)	13(36.1)	
	I	13(36.1)	7(18)	0(0)	8(21.6)	2(6.5)	1(4.0)	5(13.9)	
	R	17(47.2)	23(59)	37(94.9)	7(18.9)	22(70.9)	4(16.0)	18(50)	
*K*.*pneumonae*	S	2(18.2)	3(27.3	1(9.1)	8(72.7)	0(0)	6(100)	4(36.4)	
	I	3(27.2)	0(0)	0(0)	2(18.2)	1(9.1)	0(0)	5(45.5)	
	R	6(54.6)	8(72.73)	10(90.9)	1(9.1)	10(90.9)	0(0)	2(18.2)	
*Pseudomonas spp*.	S	0(0)	11(100)	3(33.3)	10(90.9)	0(0)	0(0)	0(0)	8(80)
	I	0(0)	0(0)	3(33.3)	0(0)	0(0)	0(0)	0(0)	1(10)
	R	12(100)	0(0)	3(33.3)	1(9.1)	8(100)	7(100)	12(100)	1(10)
**GRAM POSITIVE BACTERIA**									
*S*.*aureus*	S	9(23.1)	21(52.5)			5(12.8)	9(26.5)	10(38.5)	
	I	4(10.3)	6(15.0)			2(5.1)	5(14.7)	3(11.5)	
	R	26(66.7)	13(32.5)			32(82.1)	20(58.8)	13(50.0)	
*S*.*epidermidis*	S	2(14.3)	5(35.7)	2(15.4)	2(14.3)	3(21.43)	2(14.3)	3(37.5)	
	I	0(0)	1(7.1)	0(0)	0(0)	0(0)	1(7.1)	2(25.0)	
	R	12(85.7)	8(57.14)	11(84.6)	12(85.7)	11(78.57)	11(78.6)	3(37.5)	
*Enterococcus*	S	5(27.8)	7(18.9)			1(2.3)		21(50.0)	
	I	0(0)	13(35.1)			7(16.3)		7(16.7)	
	R	13(72.2)	17(46.0)			35(81.4)		14(33.3)	

AMC = Amoxicillin clavulanate, CIP = Ciprofloxacin, CTX = Cefotaxime, MEM = Meropenem, STX = Sulfamethoxazole/trimethoprim FOX = Cefoxitin, F = Nitrofurantoin, PTZ = Piperazillin/tazobactum. S = sensitive, R = resistant, I = intermediate.

### Multi-drug resistance

**Fig 2:**
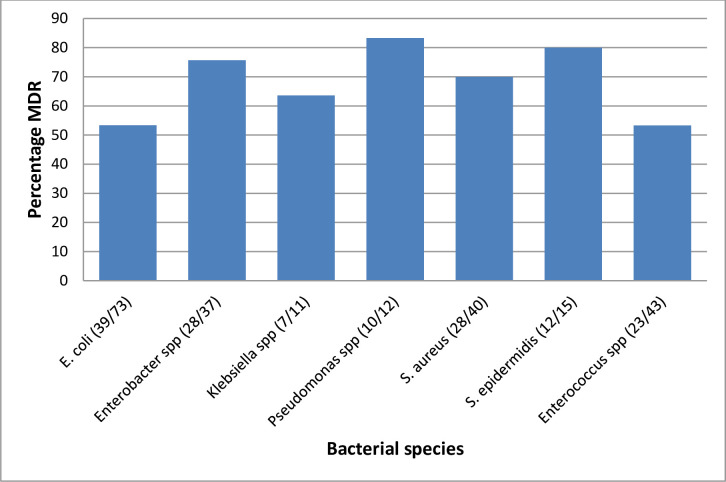
Showing multi-drug resistance of the bacterial isolate from urinary catheter tips of post-partum women.

Multi-drug resistance was high in both gram-positive and gram-negative bacterial isolates. However, gram positives 64.3(63/98) had slightly higher rates of multidrug resistance than gram-negative 63.2% (83/133). *Staphylococcus epidermidis* followed by Pseudomonas spps appeared to have the highest MDR rates (Fig 2).

### Factors associated with bacterial species diversity of indwelling urinary catheters from postpartum mothers

The bivariate analysis was done to determine the relationship between socio-demographic, obstetric, medical, and catheter-associated factors and bacterial species diversity. Urinary catheter size and catheterization duration had p-values < 0.05, 95% confidence interval, therefore, had statistical significance colonisation. Other factors were not statistically significant in this study (Tables 3 & 4).

**Table 3:** Showing association of socio-demographic characteristics and medical characteristics with bacterial species diversity.

Socio-demographic characteristics								Medical characteristics			
Characteristic	Colonisation n (%)		p-value	Characteristic	Colonisation n (%)		p-value	Characteristic	Colonisation n (%)		p-value
	Single	Multiple			Single	Multiple			Single	Multiple	
Residence				Education				Treatment of UTI			
rural	82(82)	79(76)	0.29	none	1(1.0)	3(2.9)	0.594	yes	52(52)	47(45.2)	0.331
urban	18(18)	25(24)		primary	50(50)	52(50)		no	48(48)	57(54.8)	
Age(years)				secondary	40(40)	36(34.6)		If yes, how long ago			
15–19	16(16)	23(22.1)	0.458	tertiary	9(9)	13(12.5)		<1month	16(16)	15(32.6)	
20–24	34(34)	26(25)		Family-income				1 month	4(7.7)	9(19.6)	
25–29	28(28)	29(27.9)		<290,000	43(43)	48(46.2)	0.408	>2months	28(54)	19(41.3)	0.325
>30	22(22)	26(25.0)		290,000–599,000	37(37)	30(28.8)		recurrent	4(7.7)	3(6.5)	
Marital-status				>600,000	20(2)	25(25.0)		Antibiotic use			
unmarried	14(14)	14(13.5)	0.911	Referral				yes	86(86)	90(86.5)	0.911
married	85(86)	91(86.5)		Yes	36(36)	41(39.4)	0.614	no	14(14)	14(13.5)	
Occupation				No	64(64)	63(60.6)		Duration of current antibiotics			
Formally-employed	6(6)	13(12.5)	0.214	Referral unit				one day	31(36)	28(31.5)	0.521
Informally-employed	16(16)	12(11.5)		lower health center	31(89)	9(71.8)	0.07	than1 day	55(64)	61(68.5)	
Not-employed	78(78)	79(76)		Hospital	4(11)	11(28.2)					

n = number of participants, (%) percentage of participants.

**Table 4:** Showing association of obstetric characteristics and catheter characteristics with bacterial species diversity.

OBSTETRIC CHARACTERISTICS									CATHETER CHARACTERISTICS			
Characteristics	Colonisation n (%)		P-value	Characteristics	Colonisation n(%)			P-value	Characteristic	Colonisation n (%)		p-value
	Single	Multiple			Single	Multiple				Single	Multiple	
Antenatal contacts				Vaginal					Health worker			0.563
<4	23(23)	33(32.7)		Spontaneous	3(15.8)	6(33.3)		0.226	Nurse	62(62)	67(64.4)	
≥4	77(77)	68(67.3)	0.126	Episiotomy	6(32)	2(11.1)			Doctor	22(22)	18(17.3)	
Pregnancy type				Tear	10(53)	10(55.6)			Student	12(12)	17(16.4)	
Singleton	95(95)	96(92.3)		General condition					Don’t know	4(4)	2(1.9)	
Multiple	5(5)	8(7.7)	0.431	Good	69(69)	65(62.5)		0.328	Duration of catheterization			0.024*
Parity				Poor	31(31)	39(37.5)			<2 days	32(32)	19(18.3)	
1	37(37)	30(28.9)		Length of labor					≥2days	68 (59)	85(81.7)	
2–4.	49(49)	55(51.9)	0.376	12–18 hours	7(37)	9(50)			Volume of emptying			0.235
>4	14(14)	20(19.2)		>18hours	12(63)	9(50)		0.419	Full	69(70)	65(62.9)	
Mode of delivery				Rupture of membranes					Half-full	29(30)	39(37.5)	
Vaginal	19(19)	17(16.4)		Spontaneous	25(76)	20(80)		0.080	Catheter size			0.031*
Emergency c/s	72(72)	85(81.7)	0.062	ARM	3(9)	5(20)			14	15(15)	6(5.8)	
Elective c/s	9(9)	2(1.9)		PROM	5(15)	0(0)			16	36(36)	31(29.8)	
									18	49(49)	67(64.4)	

* Characteristic with p value <0.05, C/S = Caesarean Section, ARM = Artificial Rupture of Membrane, PROM = Prelabour Rupture of Membranes.

### Logistic regression of the factors which were statistically significant with bacterial colonisation of the urinary catheter

The duration of catheterization and catheter size was statistically significant at p-value<0.05 at 95%CI (Table 5).

**Table 5:** Factors significantly associated with bacterial species diversity/ colonisation of the urinary catheter.

characteristic	Odds Ratio	Std. Err.	z	P>z	[95% Conf.	Interval]
Size						
16	2.413403	1.323431	1.61	0.108	0.823871	7.069696
18	3.858687	2.028021	2.57	0.010	1.377435	10.80956
Duration						
≥2	2.290857	0.7768027	2.44	0.015	1.178598	4.452772
_cons	0.192825	0.1108119	-2.86	0.004	0.062517	0.594742

### Knowledge and behaviours of care-takers on urinary catheter and drainage system care

In-depth interviews were conducted on 17care takers of post-partum women with urinary catheters. The mean age of participants was 32.56years with a standard deviation of 9.137, and a median age of 30years. The participants’ minimum age was 23year and the maximum age of 50 years. The majority of the participants did not have prior experience with catheterization (Table 6).

**Table 6:** Showing demographic characteristics of persons in charge of indwelling urinary catheter maintenance care (care-takers and postpartum mothers).

CODE	AGE	EDUCATION LEVEL	PARITY	PREVIOUS HISTORY OF CATHETER CARE	LENGTH OF STAY AS A CARETAKER
MHR1	23	Secondary	1	no	2
MHR2	27	primary	1	no	1
MHR3	48	primary	3	yes	3
MHR4	30	primary	1	no	5
MHR5	35	primary	1	no	1
MHR6	35	primary	3	no	2
MHR7	30	Secondary	3	no	3
MHR8	50	Secondary	2	no	3
MHR9	30	Secondary	1	no	2
MHR10	23	tertiary	1	no	3
MHR11	48	primary	3	no	3
MHR12	42	primary	3	no	2
MHR13	26	Secondary	4	no	2
MHR14	23	primary	1	no	1
MHR15	26	primary	2	no	3
MHR16	26	tertiary	1	yes	2
MHR17	33	primary	2	no	1

Codes MHR1 –Mbale Regional Referral Hospital, Respondent number 1, 2, 3.

### Health education on care of the catheter

There was limited education on urinary catheter care from the staff to the care-takers. Most care-takers reported they were taught by the health workers how and when to empty the urine drainage bags. Some care-takers also reported that health workers told them to adjust the valve and not to open it completely. A care-taker also reported that the health worker told her to empty the urine drainage bag when it’s half-full. However, some key points on catheter maintenance care such as washing hands before and after emptying the catheter, keeping the drainage bag below the level of the bladder, daily cleaning around the urethral meatus at the point of catheter insertion and intended duration of catheterization were left out.

*“Yes, I was taught about emptying the bag; the health worker told me not to open the valve completely”*

(MHR1, 23 years)

“*She was brought from operation after it was inserted*. *I was taught how to open the valve and remove urine from the bag*”

(MHR15, 26 years)

“*Today morning the doctor asked where the urine reached then I told him I was just poring then he told me it has reached around the middle before you pour*”

(MHR3, 48 years)

“*Yes they taught me how to open the valve*”

(MHR11, 48 years)

Other care-takers reported that they did not receive any information from the health workers on catheter care, but they learnt by observing their neighbours and by intuition and these would be the driving for catheter care violations.

“*No*, *I wasn’t taught but I saw what other people were doing*”

(MHR9, 30years)

“*No I wasn’t taught*. *We can look and see what the neighbours are doing*”

(MHR16, 26 years)

### Knowledge of catheter care

Care-takers reported various reasons as to why the health workers inserted an indwelling urinary catheter for their patient. These included: draining urine continuously, difficulty in movement, preparation for caesarean, allow descent of the baby. Whereas others reported they did not know why the catheter was inserted.

To continuously drain urine from the bladder.

The majority of the care-takers reported that the health workers inserted the urinary catheter to allow drainage of urine continuously.

“*To help her pass urine since she cannot go to the toilet*”

(MHR6, 35 years)

“*She had a baby in the womb but the stomach divided into two*, *one part up and another down*. *The Baby was up and bladder down*. *So they put to remove the urine*”

(MHR15, 26 years)

Care-takers also reported that the health workers inserted the urinary catheter because their patient had difficulty in movement.

“*When the patient is operated they cannot move so that why they inserted the catheter*” (MHR3, 48 years)

“*To help her pass urine since she cannot go to the toilet*”

(MHR6, 35 years)

Other care-takers reported that the health-workers inserted the urinary catheter because they were preparing the patient for a caesarean section.

*“I think to drain urine; it is because of the operation”*

(MHR14, 23 years)

*“A mother reported I was going to produce. When we came the doctor said the baby could not come out because the baby was big. He said that I was obstructed so they put the catheter to take out urine as I was going to theatre”*

(MHR7, 30years)

A care-taker reported that the health worker inserted the urinary catheter for her patient to drain urine and allow descend of the baby.

“*We came with the catheter; the doctor from the referring health centre said we need to remove the urine to allow the baby descend*”

(MHR17, 33 years)

Care-takers also reported that they did not know why the health workers inserted a urinary catheter for their patient because they were not told and they did not inquire as well.

*“The catheter was inserted before the operation but I don’t know why it was inserted*, *they did not tell me and I did not ask why*”

(MHR2, 27 years)

“*I don’t know”* (MRHR11, 48 years)

“*I don’t know why they put*” (MHR13, 26 years)

### Emptying of the urine drainage bag

Care-takers reported that they knew how to empty the drainage bag since health workers taught them while others learnt by observing their neighbours who had patients with urinary catheters. However, most of them did not take infection prevention precautions when emptying the drainage bag, such as washing hands with water and soap.

“*I know how to empty but I don’t know much*”

(MHR3, 48 years)

“*They told me to open the valve and let the urine drain*.*so when is full I empty it*. *“I wash my hand with water before and after emptying the bag*”

(MHR17, 33 years)

### Care of urinary catheters and drainage bag

Care-takers cared for urinary catheters and drainage bags daily in various ways namely, emptying the drainage bag when full or before it get full, keeping the drainage bag off the ground by putting it on the bed, putting the drainage bag in a black polyethene bag, monitoring urine output, cleaning the perineum and ensuring the urinary catheter was passing urine well. However, some aspects like regular disinfecting/ cleaning of the drainage bag /, valve and catheter tube were left out. Keeping the drainage bag on the bed was a common practice instead of keeping the drainage bag below the bladder’s level. Care-takers also reported keeping the drainage bag in a polyethene bag, which disrupts monitoring of urine out-put.

Care-takers reported that they take care for urinary catheters and drainage bags by emptying the bag.

“*When it gets full pour the urine in the basin*. *Put the urinary bag in polythene*” (MHR10, 23 years)

“*There is only one way of caring for the urinary catheter*, *which is by emptying the urine in the drainage bag*”

(MHR12, 42 years)

Care-takers also reported that they take care of the drainage bag daily by putting it on the bed, preventing the drainage bag from touching the ground.

“*If it’s full*, *I remove*, *but I don’t know much*. *It’s in a black polythene bag and on the bed*”

(MHR8, 50 years)

Other care-takers reported that they care for the urine drainage bag by tying it in a black polythene bag.

“*I tie the urine drainage bag in a black polythene bag; I remove the urine then put it back in the polyethene bag*”

(MHR9, 30 years)

A care-taker reported that she cared for the urinary catheter by monitoring the urine volume in the drainage bag.

“I *monitor the urinary bag; every time urine gets there you remove*. *I do not know what happens when it gets full or it goes back to the bladder*”

(MHR16, 26 years)

Care-takers also reported that they take care of urinary catheter by cleaning the perineum including the urethral meatus with water and a cloth another reported she adds salt and omo to water.

“*You get a polythene bag so that you poor directly in it*. *If it gets full then pour it out and put another*. *Use warm water and clothe to clean under*”

(MHR4, 30 years)

“*I get boiled water add omo*, *and salt then use it to clean the vagina and the point of insertion of the catheter tube*. *I do it after every 2 days*”

(MHR15, 26 years)

**Fig 3:**
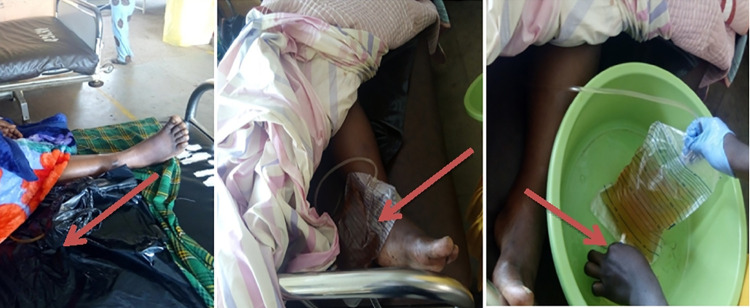
Showing some observed practices reported by the care-takers and demonstration of drainage bag emptying.

Care-takers reported that they ensured that urine was passing well. However, the urine bags were often kept in polythene bags on the bed, literally at the same level with the patient’s urinary bladder which does not allow proper urinary drainage (Fig 3).

“*The urine was pouring on the bed*. *I went and called the doctor and told her that the tube was disturbing*. *Then after a while she said we pad her and now it is passing urine well*”

(MHR6, 35 years)

### Considerations before emptying the drainage bag

Care-takers reported various considerations before emptying the urinary bag, and these included when it is full, when it is heavy before it gets full, risk of infection when it gets full, and fear that it might cause harm when it’s full and not emptied. However, they did this out of intuition. Some that had previous experience and been taught by health workers emptied the drainage bag before it go full.

Care-takers reported that they considered emptying the urinary bag when it is full as because they did not know and that’s what they do themselves. It was also observed that the care takers emptied the urine bags with bare hands.

*“When it is full”* (MHR2, 27 years)

*“If it fills up I empty, I wasn’t told by the doctors. That’s what I do myself”*

(MHR15, 26 years)

*“When it is full*, *I wait for it to fill because I don’t know”* (MHR14, 23 years)

Other care-takers reported that they emptied the urinary bag when it’s half-full.

*“When the urine in the drainage bag reaches the middle centimetres in I empty”* (MHR3, 48 years)

*“Before it gets full” (MH*R11, 48 years)

*“Before it gets full*, *cause when it gets full it will be heavy and inflict pain on the patient*”

(MHR9, 30 years)

Care-takers also reported that they empty the drainage bag when it feels heavy.

*“When it’s heavy”* (MHR1, 23 years)

Care-takers reported that they empty the drainage bag when it is full because it might cause infection if it gets full and fear of what might happen when it gets full.

*“You can leave it the urine and it goes to the stomach and causes infection”*

*(MH*R12, 42 years)

“I think it causes infection if it gets full and you don’t remove it quickly. I make sure urine doesn’t stay there for long”

*(MHR13*, *26 years*)

“*If it’s full it might pain if it’s full*. *If it is full I remove*” (MHR6, 35 years)

“*The urine looks bad for me and I don’t know what happens if it gets full*, *does it go back to the bladder or*. *Urine is dirty and when it is full*, *it’s heavy for the patient to move around with it* “

(MHR16, 26 years)

“*When it is not full because I think if it gets full it might cause her problems*”

(MHR17, 33 years)

### Challenges faced during the care of a patient with a urinary catheter

Care-takers reported facing several challenges when caring for a patient with a urinary catheter. These challenges include; the use of bare hands during emptying of the drainage bag since they have no gloves hence urine pours on their hands. The fear that the catheter may cause harm to the patient resulted in too much attention towards it, immobility since the mother became more difficult to care for, being their first experience with the care of a patient with a catheter since everything is new to them, the pain inflicted by the urinary catheter to the mother since the mothers are always complaining.

Care-takers reported that they face a challenge of using bare hands during emptying of the drainage bag since they have no gloves so urine pours on their hands.

“*I do not have gloves and then when your opening urine goes on your hands*”

(MHR3, 48 years)

“*No gloves to empty the urine so you fear*. *Sometimes the health workers are harsh and not friendly you want to ask something*, *they don’t give you time*”

(MHR16, 26 years)

Some care-takers reported that they had fear since they dint know what would happen in certain circumstances for example if the catheter fell off the bed or if the patient sleeps on the urinary tube.

“*In-case the catheter falls from the bed*, *it causes pain*” (MHR9, 30 years)

“*I don’t sleep at night; I wake up and turn the patient*. *I make sure she doesn’t sleep on the tube cause it might get out*. *The patient complains of pain*. *When the catheter is twisted the urine appears as if it’s going back and it links on the bed*”.

(MHR15, 26 years)

Care-takers also reported that care of the catheter and patient with indwelling catheters is hectic and needed a lot of attention.

“Caring for a patient with a urinary catheter is *so hectic because the patient needs more attention*”

(MHR10, 23 years)

“*Catheters need a lot of care*. *The health workers should get people to teach how to care for a catheter*”

(MHR16, 26 years)

Care-takers also reported immobility as a challenge since the mother became more difficult to care for.

“*She cannot sit-up; it’s her weakness she doesn’t want to sit-up right*”

(MHR11, 48 years)

Care-takers reported that being their first experience with the care of a patient with a catheter was a challenge since every-thing is new to them

“*It’s my first time so it has to be a challenge*” (MHR13, 26 years)

“*This is my first time*, *everything is new so am finding it difficult*” (MHR17, 33 years)

Care-takers reported pain inflicted by the urinary catheter to the mother as a big challenge since the mothers are always complaining.

“*It hurts and most people do not like it*. *It’s painful when turning so it limits movement*” (MHR2, 27 years)

*“The patient complains of pain on turning*, *sitting difficulty*, *and also fevers*”

(MHR6, 35 years)

## Discussion, study limitation, recommendation, conclusion

### Bacterial species diversity from urinary catheters of postpartum mothers

The prevalence of bacterial colonisation of urinary catheters of postpartum mothers was found to be 98%. This is in line with a similar study in Denmark where prevalence was (23/24) 96% [27]. In the same study, there was diversity in bacteria spps where positive catheters were colonised by one to two species (1.3 on average), which is similar to the findings in this study where 1–3 bacteria were isolated. However, our study findings are inconsistent with a similar study done in Colorado among 14 post-prostectomy patients, where they found that participants without systemic antibiotic therapy and duration of catheter stay of 14 days had >20 species as the estimated average [28]. This could be attributed to the difference in duration of stay being 2–3 days, the use of systemic broad-spectrum antibiotics as in The Society for Healthcare Epidemiology of America/Infectious Diseases Society of America, SHEA/IDSA practice recommendation where these factors were found to be significant [29].

All isolated microorganisms identified as shown in Fig 1 above are common in CAUTI. Except for *Pseudomonas stutzeri* a rare opportunistic pathogen commonly isolated from blood, wound, respiration tract, and urine, and *S*.*epidermidis* which has evolved as an important cause of nosocomial infection, its pathogenicity mainly due to the ability to form biofilms on indwelling medical devices increasing resistance to commonly prescribed antibiotics therefore difficult to eradicate which could predispose patients to complicated UTIs [30]. *S*. *aureus* and *S*. *epidermidis* constitute normal flora, making it difficult to distinguish an infection, contamination, and colonization. However, studies have shown these as commonly isolated gram-positive organisms from urine samples of both catheterized and none catheterized patients with UTI therefore possible human uropathogens [7,8]. These findings are in line with the findings of a study done in Nepal where 1,360 urine samples from indwelling catheter of participants reported *Staphylococcus aureus* at 52.85% as the most common gram-positive cocci isolated among patients with CAUTI [31].

Among the gram-negatives, *Escherichia coli* 97(29.6%) was the most common isolate, and *Klebsiella pneumonae* 4.3% was the least common. Like in a study in Nepal, *Escherichia coli* was the most common organism but in contrast *Klebsiella pneumonae* was the second in that study [32]. The gram-positive *Staphylococcus aureus* 68(20.7%) was the most common followed by *Enterococcus* 58(18%); these findings were similar to study among women in elective gynaecologic surgery where *Escherichia coli* was the most common gram-negative, *Enterococcus* and *Staphylococcus* were the most common gram-positive for catheters placed < 3 days [33]. A comparative study done in Egypt reported findings in line with this study where there was a difference in the prevalence of uropathogens among patients with an indwelling urinary catheter and non-catheterized patients *Enterococcus*(11.7%) and *Pseudomonas* spps (6.7%) being more common in CAUTI [34].

### Anti-microbial susceptibility of isolated bacterial species from indwelling urinary catheters of postpartum mothers

Gram-positive and gram-negative bacteria were tested on amoxicillin-clavulanate 30μg, ciprofloxacin 5μg, cefotaxime 30μg, meropenem 10μg, Sulfamethoxazole/trimethoprim 25mcg, cefoxitin 30μg, and nitrofurantoin 200mcg and piperazillin/tazobactum 110mcg. Among the Gram-negative bacteria, *Pseudomonas* spps had 100% resistance to amoxicillin-clavulanate, sulfamethoxazole/trimethoprim, cefoxitin, and nitrofurantoin and 33.3% resistance to cefotaxime while it had 100% sensitivity to ciprofloxacin followed by meropenem (90.9%) and piperazillin/ tazobactum (80%). These findings are similar to those Bangladesh, Dhaka resistance patterns of *Pseudomonas* isolates among patients with CAUTIs where they reported amoxicillin-clavulanate, cefotaxime, and sulfamethoxazole/trimethoprim to be 85.71%, 73.33%, 70% respectively. However, the resistance patterns to ciprofloxacin50% and meropenem 33% were inconsistent [35]. *E*.*coli* had a high sensitivity to cefoxitin 88.1% followed by meropenem75%, and nitrofurantoin, showed the highest resistance to sulfamethoxazole trimethoprim 92.7%, cefotaxime 85.5%, ciprofloxacin 50.7%, amoxicillin-clavulanate 45.7%. *Klebsiella pneumonae* was found to be 100% sensitive to cefoxitin, followed by 72.7% meropenem, then nitrofurantoin 36.4%. However, highly resistant to cefotaxime and sulfamethoxazole trimethoprim 90.9% followed by ciprofloxacin and amoxicillin-clavulanate. *Enterobacter* had highest sensitivity to cefoxitin 80%, followed by meropenem 59.5%, and highest resistance to cefotaxime 94.9%, followed by sulfamethoxazole trimethoprim 70.9% and ciprofloxacin 59%. Gram-negative isolates were highly resistant to sulfamethoxazole trimethoprim, cefotaxime, amoxicillin-clavulanate, and ciprofloxacin these findings are similar to a study done among catheterized patients in Nepal, high resistance patterns of isolates 83.05% to amoxicillin-clavulanate, 64.4% to cotrimoxazole, 71.18% to ceftazidime, 71.18% to ciprofloxacin [32]. Among gram-positive bacteria, *S*. *epidermidis* showed high resistance to all antibiotics with the highest resistance to amoxicillin-clavulanate, meropenem at 85.7%, cefotaxime 84.6, sulfamethoxazole trimethoprim, cefoxitin, ciprofloxacin, and highest sensitivity in nitrofurantoin 37.5%.

*Staphylococcus aureus* was highly resistant to almost-all antibiotics except for ciprofloxacin where it had 58.8% sensitivity; highest resistance was reported towards sulfamethoxazole trimethoprim 82%, amoxicillin-clavulanate at 66.7%, 58.8% were MRSA and lowest resistance to nitrofurantoin at 50%.

*Enterococcus* had 50% sensitivity to nitrofurantoin, and high resistance to sulfamethoxazole trimethoprim 81.4%, and 72.2% resistance to amoxicillin-clavulanate.

This study showed high multidrug resistance rates in both gram-negative 84(63.2%) and gram-positive 63(64.3%) bacteria species isolated from the urinary catheter tip. This could be attributed to the fact that urinary catheters provide a transient surface for binding of planktonic bacteria promoting adhesion, aggregation, and formation of micro colonies surrounded by protective secreted molecules known as extra polymeric substance matrix which later form mature biofilms thus increasing resistance to commonly used antibiotics [36]. However, MDR was slightly higher in gram-negative compared to gram-positive *Staphylococcus epidermidis*, 85.7% followed by *Pseudomonas spps*, 83.3% had the highest MDR species. These findings are in line with those reported in a study comparative study done in India where they found that 83.3% of isolates from patients with CAUTI were MDR [37]. Another comparative study done in Nepal reported an MDR of 77% among uropathogens isolated from patients with CAUTI [31].

### Factors associated with bacterial species diversity from indwelling urinary catheters of postpartum mothers

Duration of catheterization and urinary catheter size had p-values < 0.05, 95% confidence interval, therefore had statistical significance to colonization in the bivariate analysis.

Urinary catheters of participants that had stayed for greater than/ equal to 2 days were twice more likely to have multiple colonies within the catheter tip than those with urinary catheters for 1 day (P = 0.015, AOR = 2.44, 95%CI: 1.179–4.453). These findings are in line with a study done where they reported duration as the most significant factor to bacteriuria with a daily risk of acquisition of 3–7% in presence of an indwelling catheter [38].

Urinary catheters of participants with size 18 were 3 times (P = 0.010, AOR = 2.57, 95%CI: 1.377–10.80956) more likely to have multiple colonisation compared to catheters from participants of catheter size 14. This findings are in line with the Centers of Disease Control and Prevention, CDC recommendation on catheter size whereby the smaller the size the better for the patient and vice-versa [39].

### Catheter care practices and knowledge among care-takers

Care-takers knew the value of a urinary catheter. The majority of the care-takers responded correctly to why they think the health workers inserted a urinary catheter for their patient; however, a significant few reported that they did not know why the urinary catheter was inserted. Health education or teaching from health workers to patients and families is an important aspect of patient care and for a setting where care-takers are directly involved in maintenance care of a urinary catheter and drainage bag, this could significantly influence their practice positively in this study. The majority of the care-takers reported being taught by health workers on emptying of the urinary catheter, whereas some care-takers reported they were not taught on any aspect of urinary catheter care, other further added that they were just looking at what their neighbours were doing.

According to SARI and CDC guidelines on prevention of CAUTIs, catheter care, meatal cleaning with water and soap, keeping the drainage bag below the bed, hand hygiene, health education of patients and families have been recommended in routine care of the urinary catheter and drainage bag, and violation in these care practices results in an increased risk of catheter-related infection. In this study, the maintenance of urinary catheter and drainage bag care was inadequate, which would be a significant influence on the high prevalence of urinary catheter colonisation. Also, care-takers reported several challenges such as the use of bare hands while emptying the drainage bag and lack of prior exposure/knowledge which would harm catheter care practice [39,40].

## Study limitations

In this study, a culture-dependent method of species phenotypic identification was used which could have limited the bacterial diversity.

It is possible that we did not recover *Streptococcus* spps due to overnight incubation in peptone water.

All study participants had foley catheters which were none antimicrobial coated, yet the use of antimicrobial coated catheters has been associated with the lower rates of CAUTIs. Therefore the comparison in bacterial diversity in antimicrobial coated and none antimicrobial coated could not be established.

## Recommendations

The use of small gauge urinary catheters as it has been evidenced that small catheter sizes promote better patient outcomes such as reduce urethral trauma, and risk of infection as well as multiple colonisation.

Postpartum mothers and their care-takers should be educated on key aspects of catheter maintenance care cleansing the urethra area and catheter itself, keeping the drainage bag below the level of the bladder and as clean as possible, ensure hand hygiene when emptying the drainage bag/ use of gloves and avoid kinks in the tube.

## Conclusion

There was a high prevalence of catheter colonisation with bacterial spps diversity averaging 2 spps per sample despite use of broad spectrum antibiotics. The MDR rates on antibiotics commonly used to treat UTIs were high, which calls for routine culture and sensitivity for judicious use of antibiotics. Health workers practicing obstetric medicine need to pay attention to catheter sizes during catheterisation and its duration. Health education on urinary catheter care should be part of antenatal and postnatal care education.

## Supporting information

S1 Data (PDF)

S1 Raw data (XLSX)
