# Supplementary material for: Bacterial colonization, species diversity and antimicrobial susceptibility patterns of indwelling urinary catheters from postpartum mothers attending a Tertiary Hospital in Eastern Uganda
Source: PLoS One. 2022 Jan 10;17(1):e0262414. doi: 10.1371/journal.pone.0262414 (PMC8746759; doi:10.1371/journal.pone.0262414)
Supplement: S1 Data — (PDF) [file pone.0262414.s001.pdf]

QUESTIONNAIRE

STUDY TITLE: BACTERIAL SPECIES DIVERSITY AND ANTIMICROBIAL SUSCEPTIBILITY PATTERNS FROM INDWELLING URINARY CATHETERS OF POSTPARTUM MOTHERS ATTENDING MBALE REGIONAL REFFERAL HOSPITAL

I am Nakawuki Ashley Winfred, a forth year nursing student of Busitema University faculty of health sciences. I am conducting a study on bacterial species diversity, antimicrobial susceptibility patterns and associated factors amongst inpatient postpartum mothers with in-dwelling urinary catheters attending Mbale Regional Referral Hospital

I would like to ask you and your caretaker a few questions about who you are, how you progressed during labour, knowledge and behaviour during care the indwelling urinary catheter and drainage system. I will also cut the catheter tip from your catheter after removal and take for culture to identify the uropathogens and their antimicrobial sensitivity. The results from these will be analysed and shared with hospital administration which may aid management of urinary catheters postpartum mothers with indwelling urinary catheters. Your Participation in the study is voluntary and does not affect quality /treatment you receive in this hospital, and therefore you are free to withdraw from the study at any time without seeking our permission. There are no direct benefits but results will help improve quality of care of postpartum mothers with indwelling urinary catheter who are a most high risk group to catheter related infection.

NAME	THMBPRINT/SIGNATURE	DATE	TIME
MOTHER/WITNESS			
RESEARCHER			

Patient demographics

Age………………………………… Address …………………………(rural/urban)

Date of admission …………………..date of discharge ……………………..

IPNO………….. Study Number……… MUAC …….

Marital status Single ☐ married ☐ divorced ☐ widowed ☐

Occupation ……………………………………

Family income <290,000 290,000 - 599,000 >600.000

Life style

Smoking YES/NO Alcohol YES/NO

Number of sexual partner's 1/more than 1

OFFICE OF THE CHAIRPERSON
APPROVED
APPROVED DATE EXPIRY DATE
1 9 FEB 2020 1 5 MAY 2020
MBALE REGIONAL REFERRAL HOSPITAL
RESEARCH & ETHICS COMMITTEE
(MRRH- REC)

Level of education none ☐ primary ☐ secondary ☐ tertiary institution ☐

Unit level of referral Source ……………………………………………………..

Indication for referral ……………………………………………………..

Obstetric factors

Parity …………………..

Last Normal Menstrual Period …………………..

Expected Date of Delivery …………………..

Date of delivery …………………..

gestation age at delivery

Preterm(<37)	term	Postterm(>42weeks)

Mode of delivery

vaginal ☐ C-section ☐

If vaginal

I. Spontaneous ☐ episiotomy done ☐ sustained a tear ☐ vacuum/ forceps ☐

II. For long were you in labour

<6hour ☐ 6-12hours ☐ 12-18hours ☐ 18hours ☐

What was the type of pregnancy Singleton ☐ multiple ☐

What was the delivery outcome alive ☐ stillbirth ☐

Did you have high fevers during pregnacy YES ☐ NO ☐

Did you visit the Antenatal clinic in this pregnancy YES ☐ NO ☐

If yes,how many times did you visit the Antenatal clinic(ANC)

< 4 times	4-8 times	➢ 8times

Medical Factors

Where you treated for a UTI during pregnancy

Yes ☐ NO ☐

If yes ,how long ago

<1 month ☐ 1 month ☐ 2 months ☐ recurrent ☐

OFFICE OF THE CHAIRPERSON
APPROVED
APPROVED DATE EXPIRY DATE
19 FEB 2020 15 MAY 2020
MBALE REGIONAL REFERRAL HOSPITAL
RESEARCH & ETHICS COMMITTEE
(MRRH- REC)

Do you have any illnesses that you have been receiving treatment for

Diabetes mellitus ☐ HIV/AIDS ☐ others...............................

Have you been on medication of in the last two weeks prior to delivery YES/NO

If yes, which medications have you been on

....

Catheter assocaited factors

What type of the catheter was inserted? two way☐ 3 way☐

Who inserted the catheter Nurse☐ doctor☐ student☐ I dont know☐

Duration of stay of the catheter

< 2 days	2days - 3days	4 days - 5days	> 5days

When do you empty the urinary bag

Full ☐ half full ☐ quarter ☐anytime ☐

From where was the urinary catheter inserted MRRH ☐OTHER FACILITY ☐

If another facility was the catheter changed YES☐ NO☐

IF yes, after how long was it removed ....

PATIENT CHART REVIEW

Admission Diagnosis ....

Current medication and duration of use

....

....

....

Indication for catheter insertion

....

What catheter size was inserted ....

Recent hemoglobin(Hgb) concentration ....

OFFICE OF THE CHAIRPERSON
APPROVED
APPROVED DATE 19 FEB 2020
EXPIRY DATE 15 MAY 2020
MBALE REGIONAL REFERRAL HOSPITAL
RESEARCH & ETHICS COMMITTEE
(MRRH- REC)

Level of education none ☐ primary ☐ secondary ☐ tertiary institution ☐

Unit level of referral Source ……………………………………………………

Indication for referral ……………………………………………………

Obstetric factors

Parity …………………..

Last Normal Menstrual Period …………………..

Expected Date of Delivery …………………..

Date of delivery …………………..

gestation age at delivery

Preterm(<37)	term	Postterm(>42weeks)

Mode of delivery

vaginal ☐ C-section ☐

If vaginal

I. Spontaneous ☐ episiotomy done ☐ sustained a tear ☐ vacuum/ forceps ☐
II. For long were you in labour
<6hour ☐ 6-12hours ☐ 12-18hours ☐ 18hours ☐

What was the type of pregnancy Singleton ☐ multiple ☐

What was the delivery outcome alive ☐ stillbirth ☐

Did you have high fevers during pregnacy YES ☐ NO ☐

Did you visit the Antenatal clinic in this pregnancy YES ☐ NO ☐

If yes,how many times did you visit the Antenatal clinic(ANC)

< 4 times	4-8 times	➢ 8times

Medical Factors

Where you treated for a UTI during pregnancy

Yes ☐ NO ☐

If yes ,how long ago

<1 month ☐ 1 month ☐ 2 months ☐ recurrent ☐

OFFICE OF THE CHAIRPERSON
APPROVED
APPROVED DATE 19 FEB 2020
EXPIRY DATE 15 MAY 2020
MBALE REGIONAL REFERRAL HOSPITAL
RESEARCH & ETHICS COMMITTEE
(MRRH- REC)

INDEPTH INTERVIEWER GUIDE

Volunteer's statement

I have read and/or been read to and fully explained to the information sheet concerning my participation in this study and I understand what will be required if I take part. I understand that my participation is voluntary. My questions concerning this study have been answered. I understand that at any time I may withdraw from this study without giving a reason. I agree to take part in this study.

____________________	____________________	____________________
**Caretaker's name**	**Signature/thumbprint**	**Date**
____________________	____________________	____________________
**Researcher name**	**Signature**	**Date**

Topic; knowledge and behaviours of urinary catheter and drainage system care

Interviewer's guide

Why do you think the doctor/ midwife inserted a urinary catheter for this mother?

After inserting the catheter were you taught how to care for it?

In your own way how do you take care of the catheter daily?

What makes you decide to empty the urinary bag?

Are there any other measures you take to ensure that your patient doesn't get infections that would be catheter related while in the hospital?

What are the common challenges you face when caring for a mother with a urinary catheter?

What experience have you had taking care of a patient with a catheter?

OFFICE OF THE CHAIRPERSON
APPROVED
APPROVED DATE EXPIRY DATE
1 9 FEB 2020 1 5 MAY 2020
MBALE REGIONAL REFERRAL HOSPITAL
RESEARCH & ETHICS COMMITTEE
(MRRH- REC)
